# Hemorrhagic Cystitis Secondary to Adenovirus and BK Virus Infection in a Diffuse Large B-Cell Lymphoma Patient with Recent CAR T-Cell Therapy

**DOI:** 10.1155/2020/6621967

**Published:** 2020-11-27

**Authors:** Abdul Moiz Khan, Zainub Ajmal, Fatima Tuz Zahra, Ananthakrishnan Ramani, Ira Zackon

**Affiliations:** ^1^Department of Internal Medicine, Albany Medical Center, Albany, NY, USA; ^2^Department of Infectious Diseases, Albany Medical Center, Albany, NY, USA; ^3^New York Oncology Hematology, Albany Medical Center, Albany, NY, USA

## Abstract

Patients who undergo chimeric antigen receptor T-cell therapy (CAR T-cell therapy) are immunosuppressed due to multiple factors. While adenovirus and BK virus are well-known pathogens in the context of hematopoietic stem cell transplant, there are no detailed reports of these infections in the setting of CAR T-cell therapy. We describe a 70-year-old male who recently underwent CAR T-cell therapy for diffuse large B-cell lymphoma. He presented with intractable gross hematuria and dysuria. Workup revealed adenovirus viremia and viruria and BK virus viruria. He was treated for adenovirus hemorrhagic cystitis with intravenous cidofovir 1 mg/kg/day, every three days for three weeks, with good clinical response. We also discuss the mechanisms of immunosuppression in CAR T-cell therapy as well as the principles of treatment of adenovirus and BK virus infections in the immunosuppressed patient.

## 1. Introduction

Hemorrhagic cystitis (HC) is a diffuse inflammatory condition of the urinary bladder due to an infectious or noninfectious etiology resulting in bleeding from the bladder mucosa [1]. Infectious agents include bacteria, viruses (such as adenovirus and BK virus), fungi, or parasites. Noninfectious causes include medications such as cyclophosphamide, environmental toxins, and radiation [1, 2]. Prompt management of HC is vital especially in the context of immunosuppression. Chimeric antigen receptor T-cell therapy (CAR T-cell therapy) is an emerging type of immunotherapy for various hematological malignancies. Patients who undergo CAR T-cell therapy are generally immunosuppressed and prone to infections [3]. While adenovirus and BK virus are well-recognized pathogens in the context of hematopoietic stem cell transplant, only 2 cases of adenovirus viremia in CAR T-cell therapy patients have been reported by Chandorkar in a retrospective study [4]. Importantly, to the best of our literature review, there are no detailed accounts of concurrent infection with both these agents in the setting of CAR T-cell therapy.

We describe a case of HC secondary to adenovirus and BK virus in a patient with diffuse large B-cell lymphoma with recent CAR T-cell therapy. We discuss the mechanisms of immunosuppression in CAR T-cell therapy and the principles of treatment of adenovirus and BK virus infections in the immunocompromised patients.

## 2. Case Presentation

A 70-year-old male with diffuse large B-cell lymphoma presented with worsening dysuria, frequency, urgency, and gross hematuria along with mild suprapubic pain for 10 days. Hematuria was initially intermittent though it became persistent with infrequent passage of clots. The patient had failed an outpatient course of trimethoprim/sulfamethoxazole for a possible bacterial urinary tract infection. He denied fever, chills, flank pain, trauma, or weight loss. On our initial physical exam, he had a temperature of 37 C, blood pressure 120/55 mmHg, pulse 100 beats/min, respiratory rate 20/min, and oxygen saturation 98% on room air. He had prominent conjunctival pallor, a mildly distended abdomen with moderate tenderness in the suprapubic region. However, no flank or costovertebral angle tenderness, scrotal or penile swelling or tenderness, or cervical, axillary, or inguinal lymphadenopathy was noted.

The patient was diagnosed with diffuse large B-cell lymphoma in 2007 with multiple recurrences. Most recently, he had received lymphodepleting chemotherapy with fludarabine and cyclophosphamide followed by axicabtagene ciloleucel CAR T-cell therapy (2 × 10^6^ cells/kg) two months before the presentation, in December 2019. He experienced grade 2 cytokine release syndrome during the treatment which warranted therapy with interleukin-6 inhibitor, tocilizumab. He had also received multiagent chemotherapeutic regimens between 2007 and 2018 for recurrences, including an autologous stem cell transplant in 2017. Additionally, he had a 60 pack-year smoking history which he quit 5 years ago. He denied any significant travel or occupational exposures.

Initial workup revealed pancytopenia with a white blood cell count of 3.2 × 10^3^ (normal level 4–9 x 10^3^ cells/*μ*L), absolute neutrophil count 2.5 × 10^3^ (1.5–5.2 × 10^3^ cells/*μ*L), absolute lymphocyte count 0.3 × 10^3^ (1.1–3.9 × 10^3^ cells/*μ*L), hemoglobin 8.9 (13.6–16.7 g/dl), hematocrit 26.3 (40–49%), and platelet count 17 × 10^3^ (130–350 × 10^3^ cells/*μ*L). Creatinine was 1.60 (patient's baseline was 1 mg/dL) and estimated GFR 43 (normal >60 mL/min/1.73 m^2^). Urinalysis showed 1+ leukocyte esterase, negative nitrite, 3+ hemoglobin, 50–100/high power field (HPF) WBCs and too numerous to count RBCs. Urine gram stain and culture had 3+ polymorphonuclear cells but no bacterial or fungal growth. Two sets of aerobic and anaerobic blood cultures were negative. On imaging, computerized tomography (CT) scan of the abdomen and pelvis showed moderate right hydroureteronephrosis with surrounding fat stranding of the kidney and ureter along its entire course. Ureteral dilation was up to 1.1 cm and bladder wall thickening with surrounding fat stranding were seen. Adenovirus and BK virus PCR were ordered.

While the results of adenovirus and BK virus PCR were awaited, supportive care was provided with intravenous fluids, phenazopyridine, and analgesics. Patient also received intravenous immunoglobulin (IVIG) for hypogammaglobulinemia. Antibiotics were held given the low likelihood of bacterial urinary tract infection. In order to rule out obstruction based on the findings of hydroureteronephrosis, rigid cystourethroscopy, bilateral retrograde pyelogram, right ureteroscopy, bladder biopsies, and fulguration were performed. Cystoscopy revealed diffuse gross cystitis with mucosal mounding. Bilateral retrograde pyelogram demonstrated poor drainage and calyceal blunting on the right-side, hence a ureteral stent was placed, whereas the left side was normal. Meanwhile, adenovirus was detected by PCR in urine as well as serum. BK virus DNA quantitation by real time PCR in urine showed 1,300,000 copies/ml (log copies 6.11), though BK viral DNA was not detected in serum. Hypogammaglobulinemia with IgG level 288.2 (667–1485 mg/dL), IgM <20 (46–216 mg/dl), and IgA 33 (63–391 mg/dL) was also seen. Bladder biopsy, urine cytology from the right kidney, and bladder washings ruled out malignancy and showed evidence of acute inflammation. Although the patient did not show any appreciable improvement in the symptoms, the renal function gradually improved to baseline.

Once the adenoviremia/viruria and BK viruria were confirmed, and the renal function improved, the patient was started on intravenous cidofovir for adenovirus HC at a dose of 1 mg/kg/day (90 mg), three times a week, for a total of three weeks. He also received probenecid and 500 ml of normal saline before and after the administration of cidofovir for renal protection. His renal function and blood counts were closely monitored and remained stable. The patient had resolution of hematuria and marked sustained improvement of lower urinary tract symptoms within 2 weeks of completion of therapy.

## 3. Discussion

Hemorrhagic cystitis (HC) in the setting of immunosuppression can be secondary to infectious or noninfectious causes. While adenovirus viremia and viruria and BK viruria were consistent with viral HC in our case, cyclophosphamide induced HC was also considered. Early-onset HC within the first few days of cyclophosphamide administration is linked to acrolein, a urotoxic metabolite of cyclophosphamide. However, HC can develop weeks to months after treatment in patients who receive high dose of cyclophosphamide [5, 6]. Late HC is most often secondary to adenovirus and BK virus infections in the setting of immunosuppression from the cyclophosphamide rather than a direct toxic effect [6–8]. In our patient, onset of symptoms almost 2 months after cyclophosphamide administration and persistent lymphodepletion supports an indirect role of cyclophosphamide.

Patients receiving CD 19 CAR T-cell therapy are immunosuppressed for multiple reasons. Effects of malignancy itself, prior cytotoxic treatments, and lymphodepleting chemotherapy, given immediately before CAR T-cell therapy, lead to cytopenias and may disrupt mucosal barriers. CAR T-cell therapy can be complicated by cytokine release syndrome (CRS) or neurotoxicity which may necessitate the use of immunosuppressive agents such as corticosteroids and tocilizumab. In addition, CD19 CAR T-cells cause depletion of normal CD19+ B-cells which may lead to hypogammaglobulinemia [3, 9]. Our patient had all these risk factors that may have compounded the risk of adenovirus and BK virus infection. However, the persistent lymphodepletion and suppressed cellular immunity from the chemotherapy given prior to the CAR T-cell therapy likely had the highest role in development of the infections.

Human adenovirus (AdV) is a nonenveloped, double-stranded, linear DNA virus that causes a wide array of diseases in both immunocompetent and immunocompromised patients, although fulminant manifestations such as HC are more common in immunocompromised population [10]. Disseminated disease carries a high mortality rate [11]. BK virus belongs to the *Polyomaviridae* family of nonenveloped, double-stranded DNA viruses. Unlike adenovirus, BK virus infections manifest through reactivation of latent virus in the setting of immunosuppression, and clinically significant infections are less frequent in immunocompetent hosts [12, 13]. In addition to the potential of causing HC by itself, BK virus may have an augmenting role since studies have shown that BK virus increases the risk of adenovirus co-infection and contributes to enhanced Adenovirus replication [14, 15].

Treatment options for treating adenovirus HC are limited. Intravenous (IV) cidofovir appears to be the most promising drug for adenovirus infection. Close monitoring and preventive strategies are required for cidofovir-associated nephrotoxicity [11, 16]. European Conference on Infections in Leukemia (ECIL) guidelines (2011) support the treatment of human adenovirus disease with IV cidofovir along with renal protective measures such as hyperhydration and oral probenecid. The recommended dosage is 5 mg/kg/week for 2 weeks and 5 mg/kg every 2 weeks thereafter, given the lack of data on other dosage schedules [17]. However, Nagafuji described treating 16 patients with adenovirus HC following hematopoietic stem cell transplant (HSCT) with IV cidofovir (CDV) at 1 mg/kg/day, three times weekly for 3 weeks. CDV therapy cleared adenovirus from urine in 86% and led to clinical improvement in HC in 71% of the evaluated patients [18]. Yoshimura and Bordigoni have also reported encouraging results with IV cidofovir for treatment of adenovirus infections in patients with allogeneic HSCT [19, 20]. Given the acute kidney injury in our patient, a dose of 1 mg/kg/day, three times weekly for 3 weeks, was used with hyperhydration and probenecid. In addition, there are some reports of intravesical cidofovir, brincidofovir, ribavirin, and vidarabine used successfully for adenovirus infections, though further investigations are needed to establish their role [11, 21–24].

ECIL guidelines (2017) also provide a framework for the treatment of BK virus HC, which mostly relies on supportive measures such as hyperhydration, bladder irrigation, platelet transfusions as needed, and pain management. Some studies have reviewed intravenous cidofovir as an effective therapy, although there is no consensus on best dose schedule, and adverse effects mainly nephrotoxicity should be considered on a case-by-case basis [25, 26]. Overall, antiviral treatment with cidofovir remains controversial and of uncertain efficacy due to a lack of randomized controlled trials [26].

## 4. Conclusions

Patients undergoing CAR T-cell therapy are immunosuppressed and prone to pathogens including adenovirus and BK virus. Intravenous cidofovir has proven to be an effective treatment for adenovirus infections in the setting of immunosuppression. Renal function should be closely monitored and measures such as hyperhydration and probenecid should be employed to reduce the risk of cidofovir nephrotoxicity.

## Data Availability

No data were used to support this study; relevant references are appropriately cited in the manuscript.
